# Office Removal of a Subglottic Bread Clip

**DOI:** 10.1155/2013/480676

**Published:** 2013-11-27

**Authors:** David E. Rosow, Si Chen

**Affiliations:** Department of Otolaryngology, University of Miami, Miller School of Medicine, Miami, FL 33136, USA

## Abstract

*Objective*. The presence of an upper airway foreign body is an emergent, potentially life-threatening situation that requires careful but rapid evaluation and management. Organic or nonorganic material may typically be found in the pyriform sinuses or tongue base or may be aspirated directly into the tracheobronchial tree. We present here an unusual case report of a patient who accidentally ingested a plastic bread clip that was lodged in his subglottis for 15 months and report successful removal in the office under local anesthesia. *Methods*. Mucosal anesthesia was achieved with inhaled 4% lidocaine spray. Flexible laryngoscopic removal of the foreign body was then successfully accomplished. *Results*. The patient's symptoms resolved completely following removal, with no sequelae. *Conclusions*. Office removal of airway foreign bodies is feasible and can be safely done with adequate topical anesthesia, but great caution and emergency planning must be exercised.

## 1. History and Presentation

**Figure 1:**
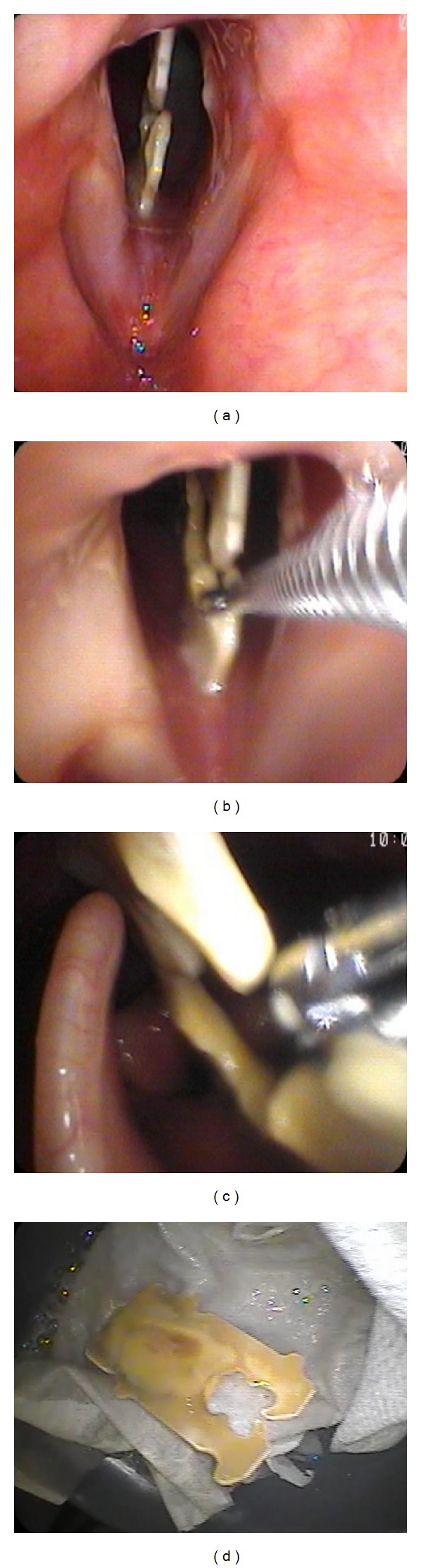
(a) Identification of bread clip wedged into subglottic airway. (b) Using a flexible endoscope with a working channel, a cupped biopsy forceps is introduced into the laryngeal introitus and used to grasp the foreign body. (c) The bread clip is easily withdrawn with the cupped forceps and ultimately removed transorally. (d) The removed foreign body, covered with purulent secretions.

A 34-year-old man was referred for laryngeal foreign body. His symptoms began 15 months prior to presentation when he felt his airway obstruct for a few seconds while eating a fish oil capsule. He developed hoarseness and globus sensation and sought consultation with an otolaryngologist at another institution. His evaluation apparently did not include laryngoscopy, and the patient was given a diagnosis of laryngopharyngeal reflux that was empirically treated with proton pump inhibitors. The patient noted that his voice actually improved over time, but the globus sensation persisted. He was not evaluated again until over a year later, when he was seen by a second outside otolaryngologist. This time, flexible laryngoscopy was done, and he was diagnosed with a laryngeal foreign body, possibly a bay leaf. On our initial office examination, a firm, linear foreign body was seen vertically bisecting the subglottis approximately 5 mm below the vocal folds, wedged between the anterior and posterior wall (Figure 1(a)). It was rigid and no movement was noted with phonation or respiration. It did not affect glottic closure, and there was no stridor or airway distress. There was no apparent granulation tissue or infection. Given these findings, approaches to removal of the foreign body was discussed with the patient. Direct laryngoscopy with microscopic removal under general anesthesia was considered, but he favored attempting awake removal with topical anesthesia only. He was consented for office removal.

## 2. Office Procedure 

The oropharynx and larynx were anesthetized with a combination benzocaine/tetracaine/butamben spray (Cetacaine) and with inhaled 4% lidocaine. A flexible distal-chip laryngoscope with working channel was used to visualize the foreign body just inferior to the glottis (Figure 1(a)). An additional 2 cc of 4% lidocaine were dripped directly onto the glottis and subglottis to ensure adequate topical anesthesia Cupped biopsy forceps were introduced through the working channel and used to grasp the foreign body, which was removed easily and atraumatically from the larynx. It appeared to be a large piece of plastic that would not fit through the nasal cavity. Thus, it was repositioned at the nasopharynx and removed transorally. Inspection revealed a completely intact 3 × 1.5 cm plastic clip from a loaf of bread. The clip was covered in purulent discharge and text on it read “Exp 5/30/11.” On further questioning, the patient admitted he might have accidentally swallowed the clip while eating a sandwich in May, 2011. The patient reported immediate relief of his symptoms, and 1-week followup confirmed complete resolution of his dysphonia and globus sensation.

## 3. Discussion

Visualization of the human larynx has changed dramatically over the past two hundred years, as improved illumination and the development of anesthesia have contributed to a central role of laryngoscopy in diagnosing and treating airway obstruction [1, 2]. Horace Green had previously described laryngeal instrumentation in the 1840s, with blind and indirect removal of foreign bodies and laryngeal lesions. Following Killian's introduction of the rigid bronchoscope in 1897, translaryngeal removal of airway foreign bodies under direct vision became increasingly popular. Schroetter reported in 1905 the removal of a tack in the bronchus of an awake child by his father Leopold, without anesthesia, via rigid upper bronchoscopy [3]. Flexible fiberoptic endoscopes revolutionized the field in the 1970s by allowing visualization of the laryngopharynx in awake, upright patients with only local anesthesia [4]. Choy et al. described transnasal fiberoptic endoscopy combined with orally introduced forceps for foreign body removal from the oropharynx and hypopharynx in 1992 [5]. Distal chip technology, which permits improved image resolution and the presence of an extra channel in the endoscope for integrated instrumentation, has further improved the clinician's ability to manipulate the airway in awake patients [6–10].

It is this most recent advance that permitted removal of the foreign body in this case without the need for general anesthesia. Many factors may influence the clinician's decision making when choosing whether to approach an airway foreign body in the operating room or in the office. The acuity of any airway obstruction, the nature and location of the foreign body, the age and overall health of the patient, and the patient's ability to cooperate with an awake procedure under topical anesthesia must all be taken into consideration. This patient was young, otherwise healthy, and able to easily tolerate office endoscopy. The stability of the foreign body was critical, as disturbing it without proper control could have led to accidental dislodgement into the trachea or bronchi, with potentially catastrophic consequences. This further underscores the need for appropriate emergency supplies if such office procedures are to be attempted, including laryngoscope, endotracheal tubes, and tracheotomy kit.

To our knowledge, this is the first report of an accidentally ingested occlupanid, as bread clips are formally known, as an airway foreign body, although review of the literature reveals several case reports of them being found in the gastrointestinal tract [11–18]. In spite of their seemingly benign nature, their teeth can hook and tear into mucosal surfaces and their plastic composition makes them radioopaque and more difficult to identify with standard imaging. As a result, some authors have called for occlupanid redesign to reduce these risks [15, 17].

Given the potential harm faced by this patient, the decision by the initial consulting otolaryngologist to forego laryngoscopic evaluation is curious at best and dangerous at worst. Globus pharyngeus, or the sensation of a lump in the throat, is often all too easy to dismiss as either a temporary or purely somatoform complaint. However, it is imperative that other pathologies of the upper aerodigestive tract be ruled out through visualization of the larynx before an empiric diagnosis of laryngopharyngeal reflux disease or conversion disorder is entertained. Remarkably, this patient did experience improvement while taking proton pump inhibitors in spite of the continued presence of the foreign body, most likely due to reduction of the overall inflammatory state of the larynx and subglottis.

## 4. Conclusions

Patient complaints of globus sensation should always be investigated with indirect laryngoscopy and not treated with empiric medical therapy. Office-based endoscopic removal of airway foreign bodies can be safely performed but should only be undertaken with proper instrumentation, topical anesthesia, and emergency supplies.
